# Different Pathways Leading to Integrase Inhibitors Resistance

**DOI:** 10.3389/fmicb.2016.02165

**Published:** 2017-01-11

**Authors:** Eloïse Thierry, Eric Deprez, Olivier Delelis

**Affiliations:** Laboratoire de Biologie et Pharmacologie Appliquée, CNRS UMR8113, Ecole Normale Supérieure de Cachan, Université Paris-SaclayCachan, France

**Keywords:** HIV-1, integrase, strand-transfer inhibitors, unintegrated viral DNA, viral resistance

## Abstract

Integrase strand-transfer inhibitors (INSTIs), such as raltegravir (RAL), elvitegravir, or dolutegravir (DTG), are efficient antiretroviral agents used in HIV treatment in order to inhibit retroviral integration. By contrast to RAL treatments leading to well-identified mutation resistance pathways at the integrase level, recent clinical studies report several cases of patients failing DTG treatment without clearly identified resistance mutation in the integrase gene raising questions for the mechanism behind the resistance. These compounds, by impairing the integration of HIV-1 viral DNA into the host DNA, lead to an accumulation of unintegrated circular viral DNA forms. This viral DNA could be at the origin of the INSTI resistance by two different ways. The first one, sustained by a recent report, involves 2-long terminal repeat circles integration and the second one involves expression of accumulated unintegrated viral DNA leading to a basal production of viral particles maintaining the viral information.

## Introduction

Although both the incidence and the number of AIDS-related deaths decreased since 1997 and 2006, respectively, AIDS remains a global health issue. Since the beginning of the pandemic, more than 35 million of people died. In 2014, the World Health Organization had estimated that 36.9 million people living with HIV, including 2.6 million of children below 15 years old. Moreover, more than 5000 new infections occur each day all over the world. Due to the high morbidity and mortality, many efforts have been made to discover efficient inhibitors of HIV replication.

After the entry of HIV into the target cell, reverse transcription occurs, coupled to both uncoating and nuclear import, leading to the conversion of viral RNA into linear double stranded viral DNA (for a review, see Campbell and Hope, 2015). During this step, high mutation frequency due to the lack of a 3′ to 5′ exonuclease proofreading allows an extensive genomic heterogeneity (Hu and Hughes, 2012).

Integrase (IN), released from the viral particle, catalyses the insertion of the resulting viral linear DNA into the host cell genome during the integration step. This process involves two consecutive reactions catalyzed by IN: the 3′-processing (3′-P) and the strand-transfer (ST) reactions (for a review, see Delelis et al., 2008b). The 3′-P consists in an endonucleolytic cleavage at each viral DNA end ensuring the positioning of viral DNA ends in the active site necessary for the ST step, consisting in their insertion in the cellular genome. Once integrated, the viral DNA, named provirus, is the starting point of the post-integrative steps from transcription to release of infectious viral particles. The integration step is crucial in the overall HIV-1 replication cycle since (i) it ensures the stability of the viral information and (ii) the provirus is described to be the sole template for an efficient viral transcription responsible in turn for the synthesis of new infectious viral particles (for a review, see Vandegraaff and Engelman, 2007). Due to its central role in HIV-1 replication, many inhibitors targeting the integration step have been developed since the end of the 1990s (for a review, see Park et al., 2015).

**FIGURE 1:**
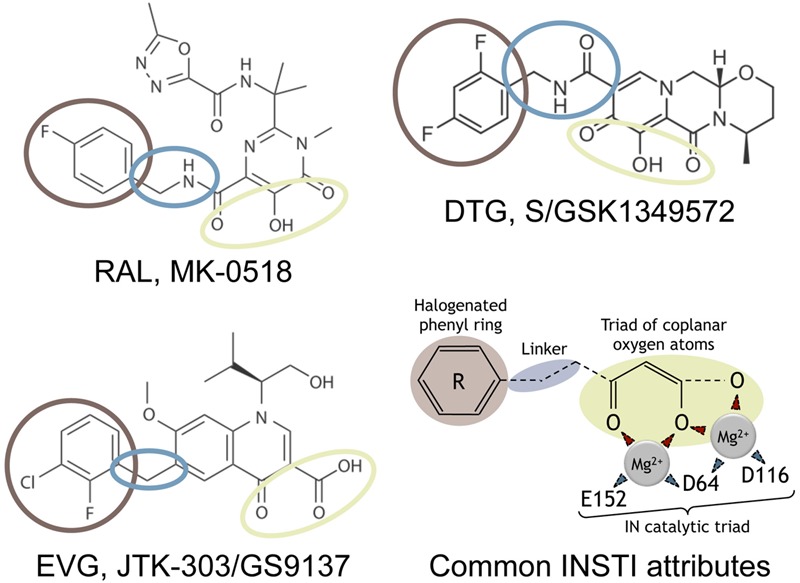
**Strand-transfer (ST) inhibitors and mechanism of action.** Chemical structures of the three integrase strand-transfer inhibitors (INSTIs) approved by FDA. The common INSTI structure indicated includes (i) a triad of coplanar oxygen atoms chelating a pair of divalent metal ions (Mg^2+^), (ii) an halogenated phenyl ring (brown) invading the pocket natively occupied by the viral DNA extremity, and (iii) a linker (blue) of variable length and flexibility which separates these two parts. The IN catalytic residues of the catalytic triad chelating the two Mg^2+^ are indicated. The two structural part of INSTIs are involved in weak interactions with IN residues, depending on the INSTI considered.

In the beginning of the development of anti-integrase inhibitors, two classes of drugs were investigated: integrase binding inhibitors (INBI) and integrase strand-transfer inhibitors (INSTI) (Zouhiri et al., 2000) (for a review, see Egbertson, 2007). These two classes were distinct from their mechanism of action. INBI inhibited the interaction of IN to viral DNA and INSTI targeted the ST step. To date, only INSTI were successfully developed to treat patients. Among these inhibitors, raltegravir (RAL) and elvitegravir (EVG) belongs to the first generation of INSTI (**Figure 1**). Unfortunately, the genetic barrier of these inhibitors has been revealed to be low, illustrated by the emergence of different pathways of resistance, thus prompting the development of second generation inhibitors. To date, dolutegravir (DTG) is the only second generation INSTI approved by the U.S. Food and Drug Administration (FDA) (**Figure 1**), and has been shown to inhibit efficiently viral resistant strains to RAL and EVG (Hare et al., 2011).

Nevertheless, it is important to note that INSTIs do not lead to the disappearance of the viral genome. Unintegrated viral DNA (uDNA) strongly accumulated in the infected cells under INSTI treatment (Svarovskaia et al., 2004). uDNA is composed of different forms of viral genomes; linear or circular. More particularly, two circular genomes can be quantified in infected cells, harboring either 1 or 2 long terminal repeat (LTRs) named 1- and 2-LTR circles, respectively. To date, uDNA is considered to be a by-product of reverse transcription without significant role in HIV-1 replication. Even if transcription of both multi-spliced and single or unspliced RNA from uDNA has been observed, only transcription of some accessory proteins, such as *nef, vpr*, and *rev* has been reported (for a review, see Sloan and Wainberg, 2011). However, uDNA accumulation under INSTI treatment could have important consequences in terms of persistence and expression of HIV-1 genomes.

In this review, we focus on the integration inhibition and in particular on different escape pathways to these inhibitors. We discuss the importance of resistance mutations but also of the role of uDNA that could explain the emergence of viral strains resistant to INSTIs compounds.

## Integrase and its Catalytic Activities

All retroviruses involved the integration step for efficient replication. Consequently, IN is a highly conserved protein and represents a common feature of the retrovirus family. HIV-1 IN is a 288-amino acids protein (32 kDa) produced by the maturation of the Gag-Pol precursor by the viral protease inside the viral particle (Asante-Appiah and Skalka, 1999). Three canonical domains can be described: (i) the N-terminal domain (amino acids 1–49) (Zheng et al., 1996; Lee et al., 1997b; Carayon et al., 2010), that contains a zinc-binding motif, favoring multimerisation of the protein (Engelman et al., 1993); (ii) the C-terminal domain (amino acids 213–288) that is mainly involved in the stability of the IN-DNA complex; and (iii) a catalytic or core domain that displays a stable dimeric organization (Goldgur et al., 1998; Maignan et al., 1998; Chen et al., 2000; Laboulais et al., 2001; Wang et al., 2001; Cherepanov et al., 2005a; Hare et al., 2010a) encompassing the three highly conserved acids residues constituting the catalytic triad: Asp64, Asp116, and Glu152; referred as the DDE motif responsible of the chelation of the divalent metal ions Mg^2+^ or Mn^2+^ (Delelis et al., 2008b; Hare et al., 2012). This catalytic triad is involved in all IN activities as described below.

It is important to note that the IN catalytic activities are ensured through the catalytic triad and the multimerisation of the protein. Previous reports demonstrate the importance of the multimeric state to ensure the proper catalytic activities of the protein (Engelman et al., 1993; van den Ent et al., 1999). For example, Zn^2+^ enhances the Mg^2+^-dependent activity of IN by promoting its multimerisation and cooperativity of DNA-binding (Lee et al., 1997a; Cherepanov et al., 2005a). Several independent studies reported two distinct oligomeric states responsible for IN activity: (i) dimers of IN responsible of the 3′-processing activity (Deprez et al., 2000, 2001; Faure et al., 2005; Guiot et al., 2006; Baranova et al., 2007; Delelis et al., 2008a) and (ii) dimers of dimers involved in the ST reaction (Li and Craigie, 2005; Li et al., 2006).

*In vitro*, the binding of retroviral INs on their cognate substrate, i.e., the LTR extremity, does not seem to require a specific sequence. Nevertheless, Prototype Foamy virus 1 (PFV-1) IN, the model of HIV-1 IN from a structural point of view, displays a higher affinity for its cognate sequence compared to a random sequence (Delelis et al., 2008a). This observation could be explained by a higher solubility of PFV-1 IN compared to HIV-1 IN. Indeed, even if the oligomeric state depends on IN concentration, less aggregates was found in PFV-1 IN purification compared to HIV-1 IN. This could favor a better fixation of IN monomers and/or dimers to the detriment of aggregates in the case of PFV-1 IN and then increase the number of specific IN/DNA complexes requiring the correct positioning of IN on its sequence (Delelis et al., 2008a). *In vitro*, the monitoring of the IN binding onto oligonucleotide (ODN) mimicking the viral DNA have shown cooperativity mediated by the Zn^2+^ motif in the N-terminal domain only in the presence of the specific ODN and Mg^2+^ (Carayon et al., 2010). The highly conserved terminal 5′-CA dinucleotide is critical for the 3′-P activity (Leavitt et al., 1992; Brown et al., 1999), but the length of the viral attachment sequence (att) involved in the formation of the IN competent complex is not precisely determined. First, previous studies have shown that the terminal 12 base pairs were involved in HIV-1 IN/DNA specific contacts (Masuda et al., 1998). A recent study based on the PFV-1 IN structure complexed to its cognate sequence, revealed specific contacts between IN and DNA on the last 10 nucleotides from the LTR extremity, and non-specific interactions on the 17 last nucleotides (Hare et al., 2010a).

Four activities are ensured by IN. 3′-P and ST activities are the two main activities described both *in vitro* and *in vivo*. Both reactions require the full-length protein, the integrity of the catalytic triad and a metallic cation (Mn^2+^ or Mg^2+^) (Delelis et al., 2008b). 3′-P reaction, corresponding to a nucleophilic attack by a water molecule on the viral DNA, is highly specific and strictly requires the CA-dinucleotide sequence, just before the terminal GT dinucleotide that is removed during the reaction (Esposito and Craigie, 1998). Thus, this reaction ensures the maturation of both viral DNA ends necessary for the subsequent reaction, ST. In an infected cell, the 3′-P reaction is efficient since the linear DNA from the reverse transcription is immediately cleaved by IN after its formation (Munir et al., 2013). Interestingly, in the case of PFV-1, the 3′-P occurs only on the 3′-LTR whereas the 5′-LTR is not involved in this process (Juretzek et al., 2004) (for a review, see Delelis et al., 2004). The consequence of this asymmetrical maturation on the overall integration process catalyzed by PFV-1 IN remains elusive.

The resulting 3′-processed DNA is then used as a substrate for the integration process. During this reaction, the nucleophilic agent is constituted by the 3′-OH of the 3′-processed DNA end (Li and Craigie, 2005). ST reaction, performed preferentially by a dimer of IN, corresponds to the integration of one DNA extremity. This half-site ST reaction can be easily recorded *in vitro* using ODN or long substrate DNA (Sinha et al., 2002; Sinha and Grandgenett, 2005; Li et al., 2006; Benleulmi et al., 2015). Concerted integration involves the integration of two viral DNA extremities in the same location leading to the 5-bp duplication (in the case of HIV-1) of the sequence flanking the integration site, and is catalyzed by a tetramer of IN (dimer of dimer) (Lesbats et al., 2008; Benleulmi et al., 2015). This overall process actually corresponds to the full-site integration process that occurs *in vivo* and can be performed *in vitro* by recombinant IN and purified PIC (Faure et al., 2005; Sinha and Grandgenett, 2005).

A third activity of IN, requiring the full length protein, has been identified by several independent groups and consists in a specific endonucleolytic activity of IN onto a short ODN mimicking the palindromic sequence found at the LTR-LTR junction of 2-LTRc (Delelis et al., 2005, 2007; Shadrina et al., 2014; Zhang et al., 2014). This reaction occurs symmetrically on the two DNA strands, at the CA position involved in the 3′-P reaction. This reaction is also highly specific using a plasmid harboring the LTR-LTR junction (Delelis et al., 2007). Importantly, this activity has been recently reported to occur during HIV-1 replication (Thierry et al., 2015). Finally, disintegration can be considered to be the reverse of the ST reaction. However, this reaction was only observed *in vitro* and can be performed by IN lacking the N-terminal or the C-terminal domain, in contrast to the three above-mentioned activities (Gerton and Brown, 1997; Leh et al., 2000; Zhang et al., 2013).

Integrase interacts with numerous host cell proteins, such as HSP60, BAF (Barrier-to-autointegration factor), HMG I(Y), INI-1 (Integrase interactor 1), and Gemin2 (Kalpana et al., 1994; Li et al., 2000; Parissi et al., 2001; Lin and Engelman, 2003; Hamamoto et al., 2006; Mathew et al., 2013). These partners modulate HIV-1 replication by direct or indirect interactions with IN, not exclusively at the integration step but also at post-integrative steps in the case of INI-1 (Mathew et al., 2013). The main cellular partner of HIV-1 IN is LEDGF/p75 (for a recent review Debyser et al., 2015). LEDGF/p75 interacts directly with IN and has a major role in the integration efficiency. LEDGF/p75 greatly enhances both 3′-P, ST and concerted viral integration (Cherepanov et al., 2003; Llano et al., 2006; Botbol et al., 2008; Engelman and Cherepanov, 2008; Maillot et al., 2013; Fadel et al., 2014). Moreover, LEDGF/p75 has been reported to have major role in post-integration step by silencing expression of the provirus by maintaining histone occupancy at the HIV-1 promoter thanks to its interaction with Spt6 and Iws1 (Gerard et al., 2015). Due to the central role of LEDGF in the overall replication process, many efforts are under investigations to impair the LEDGF/p75 interaction.

## Targeting HIV-1 IN

Due to the crucial role of IN in HIV-1 replication and considering the absence of cellular counterpart, IN represents an important target to treat HIV infection. Two main strategies are investigated to develop inhibitors: (i) catalytic inhibitors targeting 3′-P or ST reaction and (ii) non catalytic inhibitors targeting IN/LEDGF interactions.

Inhibitors targeting the catalytic site or other regions involved in the binding of DNA substrate were the first to be developed. This family includes nucleic acids or nucleotide-based inhibitors (Mazumder et al., 1997; Pinskaya et al., 2004), peptides (Sourgen et al., 1996), small organic polycyclic compounds (Robinson et al., 1996; Deprez et al., 2004) and impair the binding of IN to the viral DNA end. However, only inhibitors that preferentially or specifically target the ST reaction have reached clinical use, belonging to the INSTI family. To date, from a chemical point of view, nine classes can be determined among INSTIs, based on their scaffolds (for a review, see Li et al., 2015). RAL, the first anti-integrase inhibitor approved by FDA in 2007 (Grinsztejn et al., 2007; Summa et al., 2008), was followed by EVG in 2012 and DTG in 2013, the latter belonging to the second generation of INSTI (for a review, see Serrao et al., 2009) (Hare et al., 2011). All these compounds target the IN/DNA complex and not IN alone (Espeseth et al., 2000).

Raltegravir, EVG, and DTG share a structure in two moieties joined by a linker (**Figure 1**). The first one contains three oxygen atoms chelating the metallic cations indispensable for the IN catalytic activities. The second one, the halogenated benzyl group, interacts with the G:C base pair of the viral DNA end, preceding the terminal adenine, and with residues 145 and 146 of IN. This interaction leads to the displacement of the terminal adenine of the 3′-processed DNA from the active site. Furthermore, INSTI binding competes with the binding of the target DNA in the active site (Espeseth et al., 2000; Maertens et al., 2010) (for a review, see Engelman and Cherepanov, 2012).

Raltegravir and EVG, the latter being given with a pharmacokinetic booster, such as cobicistat, demonstrated their strong efficacy to counteract HIV-1 replication even in highly therapy antiretroviral-experienced patients (Steigbigel et al., 2008; Marchand, 2012). This efficacy is similar for HIV-2 in the case of RAL (Charpentier et al., 2011; Ni et al., 2011).

However, RAL and EVG treatments lead to resistance caused by mutations in IN gene, involving for instance the Q148/G140 and N155/E92 pathways (Cooper et al., 2008; Malet et al., 2008; Shimura et al., 2008; Delelis et al., 2009). A third pathway involving the Y143 residue has been specifically described for RAL (da Silva et al., 2010; Delelis et al., 2010). While IN polymorphism has low impact on INSTI susceptibility when no associated to a resistance mutation (Van Baelen et al., 2008; Low et al., 2009), some mutations such as S119R have been shown to increase the resistance to INSTIs when combined to the primary mutations Y143C, Q148H, and N155H (Hachiya et al., 2015). Otherwise, secondary non-polymorphic mutations are selected according to the observed resistance pathway (Rhee et al., 2008). Primary resistance mutations confer a selective advantage explaining their emergence (Quercia et al., 2009) but can be associated with different IN activity defects (Marinello et al., 2008; Delelis et al., 2009), depending on the nature of the residue substituted. For example, Y143R/C mutations lead to a similar decrease in 3′-P activity while the ST activity of the Y143C mutant is more reduced compared to the Y143R mutant (Delelis et al., 2010). Secondary mutations have been described to increase the INSTI resistance, such as the E92Q mutation associated with the Y143 or N155H pathways, or to restore the defect of activity due to the primary mutation, exemplified by the G140S that leads to a recovery of the activity of the Q148H mutant (Fransen et al., 2009; Huang et al., 2013). Interestingly, some mutations in the reverse transcriptase or protease can compensate the decrease of IN activity due to primary mutations (Buzon et al., 2010a), highlighting the functional cooperation between IN and other viral proteins.

Similar susceptibilities between PFV-1 and HIV-1 INs, coupled with the PFV-1 structure, allowed to obtain information for a better comprehension of mechanisms involved in resistance (Valkov et al., 2009; Hare et al., 2010a).

Structures of PFV-1 intasome, complexed with INSTIs, confirmed the importance of the halogenated benzyl group, as well as the three oxygen atoms allowing complete octahedral coordination of both Mg^2+^ in the active site (Hare et al., 2010a,b, 2011).

Raltegravir interaction was observed with the Y212 residue of PFV-1 (equivalent to the Y143 in HIV-1), its oxadiazole ring laying π interactions with the aromatic ring of tyrosine. Loss of this interaction in the mutants of the Y143 pathway can explain emergence of resistant mutants belonging to the Y143 pathway (Hare et al., 2010b). Structural studies highlight that, in the context of R224H mutant of PFV-1 (equivalent to the N155H mutant in HIV-1), an interaction occurs between the histidine and the phosphate group of the terminal adenine (in terminal 3′ position of the processed viral DNA). RAL was shown to be inefficient to abolish this distinctive interaction thus explaining the resistance of the N155H mutant (Hare et al., 2010b).

Finally, the resistance of the Q148H mutant was explained by the need for large and energetically unfavorable conformational changes to allow RAL binding (Hare et al., 2010b). The rapid emergence of pathways involved in resistance of RAL and EVG demonstrated that both RAL and EVG have a low genetic barrier.

## Second Generation INSTIs

To impair these resistance pathways described previously, INSTIs belonging to the second generation, such as DTG, have been developed. DTG has proven its efficacy in naive patients when combined with nucleotide reverse transcriptase inhibitors (NRTIs) with non-inferiority efficiency compared to RAL (Raffi et al., 2013; Walmsley et al., 2013). Furthermore, the VIKING trial (Eron et al., 2013; Castagna et al., 2014; Akil et al., 2015) reported the efficacy of DTG when administrated to patients with virological failure due to the emergence of primary mutations conferring resistance to RAL and EVG. However, the same study reported that DTG was less efficient concerning mutants of the Q148 pathway (Eron et al., 2013; Castagna et al., 2014; Akil et al., 2015). To date, no pathway leading to DTG resistance has been highlighted by *in vitro* selection. Only some mutations in the C-terminal domain of IN have been reported to confer a moderate resistance to DTG (Anstett et al., 2015; Cutillas et al., 2015). The study of the susceptibility to DTG of the mutants resistant to the first generation of INSTI confirmed its highest genetic barrier (Underwood et al., 2012; Canducci et al., 2013). The intrinsic stability of DTG onto the IN/DNA complex mainly explains the higher efficacy of DTG compared to RAL and EVG. Indeed, the dissociation half-time for RAL and EVG are 8.8 and 2.7 h, respectively, compared to 71 h for DTG. Structural studies using PFV-1 reported that DTG binding is similar to other INSTIs belonging to the first generation (Hare et al., 2011), i.e., that the three coplanar oxygen atoms allow the chelation of Mg^2+^ cations, while the halogenated group competes with the binding of the 3′-processed end (Hare et al., 2010a). However, some features characterized the DTG binding to the IN/DNA complex. DTG is characterized by a straighter structure compared to RAL and EVG which enables the deeper penetration of this compound into the space released by the movement of the terminal adenine of the 3′-processed DNA leading to more stable interactions with the adjacent cytidine (Hare et al., 2011). This explains why mutations at position G118 confer resistance to DTG (Malet et al., 2014; Munir et al., 2015). Moreover, van der Waals interactions between the two-fluoro atom of DTG and the Cγ and Cδ atoms of the E221 residue (equivalent to the E152 in HIV-1) (Hare et al., 2011) and between the four-fluoro atom of DTG and the C6 of the guanine are closer to those involved with RAL and EVG. In a general manner, structural study indicates a greater adaptability of DTG, compared to RAL and EVG, to the structural modifications induced by the mutants from the first INSTI generation (Hare et al., 2011; DeAnda et al., 2013).

Other second generation INSTIs are under development. Cabotegravir (GSK1265744), showing a similar structure to DTG, is under phase II clinical testing and was shown to be efficient in the reduction of the viral load (for a review, see Spreen et al., 2013). *In vitro* assays demonstrated its efficiency to impair replication of resistant mutants from the first generation with the notable exception of the mutants belonging to the Q148 pathway (Yoshinaga et al., 2015). The intrinsic properties of cabotegravir allow formulation of injectable nanosuspension in order to develop a long-acting antiretroviral treatment (Spreen et al., 2013).

## Non-Catalytic IN Inhibitors

Anti-IN inhibitors were first focused on the inhibition of catalytical activities. However, due to the emergence of viral strains resistant to INSTIs, compounds with another mode of action were developed. Interestingly, the IN binding domain (IBD) over-expression leads to a decrease in HIV-1 integration efficiency by a competition with the endogenous LEDGF (De Rijck et al., 2006). The essential role of LEDGF in HIV-1 integration mentioned above and the determination of the structural determinants involved in IN/LEDGF interaction allow to define a therapeutic target (Cherepanov et al., 2005a,b; Emiliani et al., 2005; Christ and Debyser, 2013). Several peptides, derived from the IBD, were efficient to impair IN oligomerization and thus prevented its catalytic activities (Hayouka et al., 2007; Al-Mawsawi et al., 2008). Moreover, inhibition efficiency increased when cyclic peptides were used (Hayouka et al., 2010a,b). Other peptides were developed to specifically impair IN oligomerization belonging to the “shiftides” family (Kessl et al., 2009; Maes et al., 2012). A similar approach, based on the design of peptides impairing IN/LEDGF interaction but targeting LEDGF, was employed. Expression of these peptides using lentiviral vectors was efficient to inhibit viral replication without cellular toxicity (Desimmie et al., 2012).

Screenings of existing or virtual chemical libraries, as well as the development of compounds based on the IN/LEDGF interface, have been performed (Du et al., 2008; Hou et al., 2008; De Luca et al., 2009, 2010; Christ et al., 2010, 2012; Fan et al., 2011; Peat et al., 2012). Several molecules have been shown to be efficient under the micromolar range (EC50 < 100 nM) (Christ et al., 2012; Fader et al., 2014) and are now referred to as LEDGINs, NCINIs (non-catalytic site IN inhibitors) or ALLINIs (Allosteric IN inhibitors). A common feature of these compounds is the presence of an acetic acid mimicking the D366 residue of LEDGF, the latter involved in the IN interaction using the D170, H171, and T174 residues (for a review, see Demeulemeester et al., 2014). These inhibitors have been described to display three modes of action. First, they inhibit HIV-1 integration by impairing the IN/LEDGF interaction (Christ et al., 2010). Second, they can favor the formation of inactive IN multimers (Desimmie et al., 2013; Le Rouzic et al., 2013). By enhancing IN multimerization, LEDGINs interfere with IN catalytic activities in an allosteric manner, leading to 2-LTR circles accumulation similarly to RAL treatment (Hayouka et al., 2007). Finally, they are also able to target the post-integrative steps leading to inactive viral particles formation with aberrant capsids (Balakrishnan et al., 2013; Sharma et al., 2014). Interestingly, only inhibitors targeting integrase catalytic activities lead to 2-LTR circles accumulation, which is not observed with NCINIs such as GS-B (Al-Mawsawi et al., 2008). These results highlight that 2-LTR circles accumulation is not systematically observed when HIV-1 integration is inhibited, but depends on inhibition of IN catalytic activities. The lack of 2-LTR circles accumulation after NCINIS treatment could be explained by their impact on viral DNA synthesis.

Among the NCINIs, the compound BI-224436 is under phase I clinical testing after showing its efficacy in *in vitro* assays, in infected cells and in experiments on animals (Fenwick et al., 2014). This compound has shown no cross-resistance with RAL and EVG (Fenwick et al., 2014). The resistant mutants G140S/Q148H are efficiently inhibited by BI-224436 (Fenwick et al., 2014). Conversely, the resistant mutants selected *in vitro* by BI-224436 (for example A128N) were sensitive to RAL and EVG. These promising results obtained by these inhibitors and the fact that there is no cross-resistance with INSTIs provides an opportunity to use them in combination for future treatments. However, due to differences in the residues of HIV-2 IN involved in the interaction with LEDGF and targeted by these compounds, their development has to be specifically investigated for HIV-2 to overcome this natural resistance (Christ et al., 2010; Desimmie et al., 2012).

Emergence of IN mutations, leading to INSTI resistance, constitutes the classical way for the virus to escape when INSTIs are used. However, recent clinical trials involving DTG treatment in naïve patients did not report any resistance mutation in the cases of virological failure (Raffi et al., 2013; Walmsley et al., 2013; Molina et al., 2014). This observation suggests another pathway of escape used by the virus to replicate under INSTI treatment. One hypothesis is based on recent studies underlying the roles of unintegrated HIV genome, accumulated under INSTI treatment.

## Unintegrated Viral DNA

As mentioned previously, integration of the viral genome is a central step in the HIV-1 replication life cycle since it ensures the stability of the viral information and efficient transcription. The provirus is considered to be the sole template for HIV-1 expression. It is important to note that, impairing HIV-1 integration does not lead to the disappearance of HIV-1 genomes in infected cells but to the formation of uDNA instead of integrated DNA (Chun et al., 1997; Sharkey et al., 2000). Multiple forms of uDNA could be detected as linear (issued from the reverse transcription step and precursor of the provirus) or circular forms. Two circular forms exist, harboring 1 or 2-LTR, and are called 1 and 2-LTR circles, respectively. 1-LTR circles (1-LTRc) and 2-LTR circles (2-LTRc) are mainly detected in the nucleus of the infected cell. The origin of such circular forms is diverse but the common point is that these circular forms derived from linear viral DNA and are found in the nucleus of infected cells (Munir et al., 2013). The circular nature confers them a greater stability compared to the linear viral DNA, the latter being quickly degraded. They are only diluted by cell division (Sharkey et al., 2000; Butler et al., 2002; Pierson et al., 2002; Munir et al., 2013). Due to their apparent stability, they were used as a marker of recent infections even if they can persist for a long time in cells with a weak division rate such as macrophages (Sharkey et al., 2000; Gillim-Ross et al., 2005).

1-LTRc are mainly due to recombination between each LTR by homologous recombination (HR) despite several conflicting reports. Viral extremities are recognized by the MRN complex (MRE11/RAD50/NBS1), activated by the ATM pathway as soon as the reverse transcription step occurs, and then supported by the proteins of the HR pathway (Kilzer et al., 2003). However, 1-LTRc quantification in cells deficient in MRE11 protein did not result in a decrease of 1-LTRc amount (Sakurai et al., 2009). Indeed, a significant proportion of 1-LTRc has been shown to be generated in the cytoplasm during reverse transcription (Munir et al., 2013).

2-LTRc are formed by circularization of linear DNA by the non-homologous end-joining (NHEJ) pathway (Kilzer et al., 2003). A peculiar feature shared by many retroviruses is the presence of a palindromic sequence at the LTR-LTR junction (Delelis et al., 2005, 2007). The amount of 1-LTRc can reach 20–30% of the viral genome whereas 2-LTRc amount is quite low in wt infection (2–5% of total viral DNA) (Munir et al., 2013). uDNA has been considered for a long time as a by-product of reverse transcription with no significance in the overall process of HIV-1 replication (Sloan and Wainberg, 2011). However, it is important to note that inhibition of HIV-1 IN catalytic activities lead to an accumulation of uDNA and more particularly circular viral DNA forms (Munir et al., 2013). While the 1-LTRc representativeness can reach more than 50% of total vDNA, the greater increase is observed with the amount of 2-LTRc, that can be increased by a 10-fold factor (Munir et al., 2013). Consequences of such accumulation are yet poorly described.

Despite their efficiency to inhibit integration, it is worthy to note that viral replication still occurs under INSTI treatment, raising the question of the viral genome originating this residual replication.

## Different Ways to Bypass INSTIs Effects

### uDNA Expression

The role of uDNA expression in the HIV-1 cycle is still a matter of debate. Indeed, uDNA displays the same genomic organization as the provirus. However, all reports agreed that uDNA expression is weaker compared to the provirus. Interestingly, a recent report clearly demonstrates that viral production could be detected from uDNA after reactivation of resting CD4^+^ T cells (Chan et al., 2016). Depending on the experimental settings, expression of uDNA is comprised between 10 and 70% of the proviral expression (Stevenson et al., 1990; Iyer et al., 2009). Transcription from uDNA leads to the synthesis of unspliced and spliced viral RNAs but spliced RNAs are found in a greater amount (Wu and Marsh, 2001, 2003; Kelly et al., 2008). The low amount of Rev protein, that is essential for the late replication stages, has been shown to contribute to the mechanisms leading to a weaker replication from uDNA compared to proviral DNA. Indeed, infections of cells expressing Rev lead to an efficient replication from uDNA (Sloan et al., 2011). However, uDNA expression occurs during infection of different cell lines using INSTIs or an IN catalytic mutant. In this case, viral gene expression of early genes such as Tat can be highlighted by the transcriptional activity of Tat on LTRs (Wu and Marsh, 2001; Gelderblom et al., 2008; Kelly et al., 2008). Among proteins translated from fully spliced mRNA, only Nef was directly observed (Sloan et al., 2011). Furthermore, it has been clearly demonstrated that, under specific conditions, HIV-1 replication could be evidenced without integration (Gelderblom et al., 2008; Trinite et al., 2013). More particularly, Chan et al. (2016) demonstrate that, in resting CD4 T cells, uDNA leads to the production of infectious viral particles. Several parameters influence uDNA expression. For example, in conditions where integration is impaired (catalytic mutant IN or INSTI treatment), Vpr protein enclosed within viral particles promotes uDNA transcription leading to Tat expression (Trinite et al., 2013). Furthermore, HDAC inhibitors lead to an increase of uDNA expression, suggesting a chromatin organization of uDNA (Kantor et al., 2009). However, the detection of transcripts does not ensure the presence of the viral proteins since a post-transcriptional control could be involved, as suggested by a controversial report studying SLFN11 (Li et al., 2012).

Expression of uDNA is mainly due to circular genomes. Indeed, linear DNA can be excluded due to its weak stability in the cell. Both 1-LTRc and 2-LTRc have been shown to lead to infectious viral particles when transfected into HeLa cells even with a low efficiency compared to the proviral DNA (Cara et al., 1996). Although a specific type of mRNA transcribed from 2-LTRc has been detected (Brussel and Sonigo, 2004), it has been reported that uDNA expression from 1-LTRc is stronger than from 2-LTRc (Cara et al., 1996). A recent report sustains this observation since uDNA expression is similar after infection of cells deficient in 2-LTRc formation (Thierry et al., 2016). The precise contribution of these two circular forms to uDNA expression needs to be further investigated.

**FIGURE 2:**
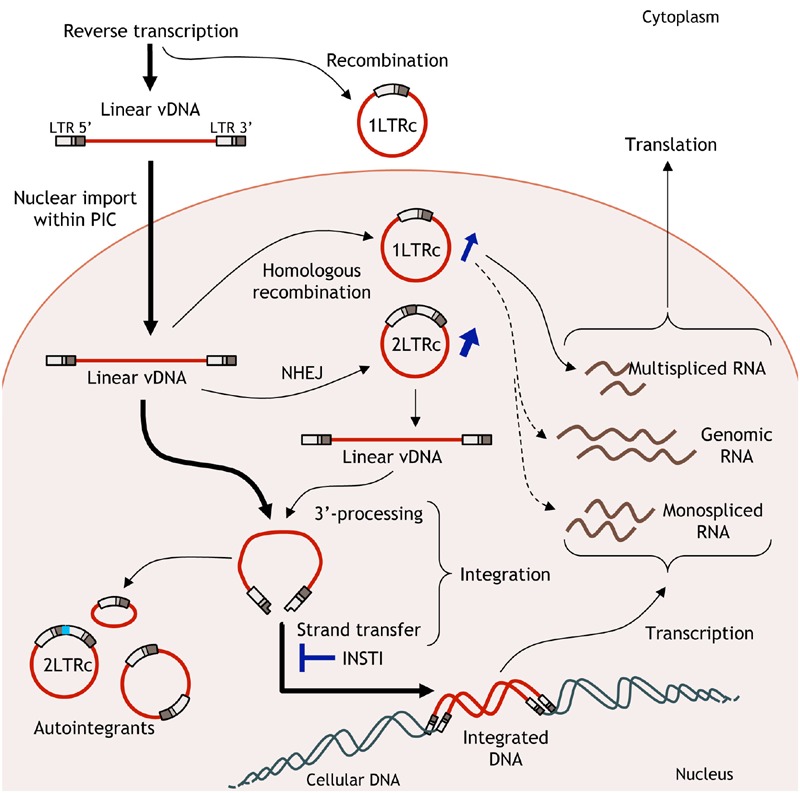
**Fate of unintegrated viral DNA (uDNA).** Linear viral DNA from the reverse transcription step can have several behaviors. Linear DNA is integrated in the host cell genome or circularized leading to 1 or 2-long terminal repeat (LTR) circles. Basal transcription from uDNA could lead to the production of infectious viral particles bypassing the effects of ST inhibitors.

Due to the strong accumulation of circular forms under INSTIs treatment, uDNA could play an important role in viral expression and could lead to a weak viral particle synthesis bypassing the INSTIs treatment (**Figure 2**). Despite their great efficiency to inhibit the integration step, INSTIs are unable to impair the late stages of the viral replication cycle, and thus they can’t prevent new infections. Clinical studies have not only shown that low-level viremia can persist in patients with undetectable plasma HIV RNA (Maldarelli et al., 2007) including patients treated with RAL-based regimens (Baroncelli et al., 2015), but also that RAL intensification is unable to suppress this persistent residual viremia and is associated with an increase in circular uDNA (Dinoso et al., 2009; Buzon et al., 2010b; Gandhi et al., 2010; McMahon et al., 2010; Hatano et al., 2011; Vallejo et al., 2012). An evolution of HIV-1 envelope sequences despite potent antiviral therapy has previously been shown (Gunthard et al., 1999; Martinez et al., 1999) with the emergence of a NRTI resistance mutation (Martinez et al., 1999). Such ongoing basal replication could occur from uDNA and, taking advantages of the inability of INSTIs to impair new infections, mutations leading to INSTI resistance could occur during reverse transcription in newly infected cells.

### INSTI Reversibility

As mentioned previously, inhibition of integration by INSTIs is dependent from the residence time of the compounds on the complex formed by the drug, IN and viral DNA. Stability of the INSTI on the complex depends on the compounds studied, since different discordant half-life have been described for RAL, EVG, and DTG (8.8, 2.7, and 71 h, respectively) (Hightower et al., 2011).

It has been recently reported that removal of RAL from cell medium until 72 h post-infection leads to viral resumption mediated by *de novo* integrated events (Thierry et al., 2015). This viral resumption was due to the cleavage of the LTR-LTR junction of 2-LTRc followed by their integration in the host cell genome indicating that 2-LTRc, accumulated under INSTIs treatment, can be used as a substrate for integration process. Moreover, the observation of the 5 bp duplication associated with these integration events, considered as HIV-1 integrase mediated integration hallmark, underlined the specificity of these events. These results also highlight the biological relevance of the endonucleolytic *in vitro* activity of IN onto the LTR-LTR junction, in the specific context of 2-LTRc accumulation caused by INSTI treatment. These data highly suggest that 2-LTRc can be considered as a back-up molecule.

Based on this result, one can speculate that, infection of non-dividing cells such as quiescent CD4-T cells can lead to 2-LTRc accumulation under INSTI treatment. Indeed, the presence of uDNA in macrophages infected with a non-integrative virus has been detected up to 30 days post-infection (Kelly et al., 2008). After cellular activation, 2-LTRc could be used as a substrate for integration. To explain how this activity could be used in the virological context several hypothesis can be advanced. The first one is that the LTR-LTR junction maintained IN in an active form due to the rather stability, in non-dividing cells, of uDNA. The second one, supported by several reports, involves a faint viral production from uDNA probably due to its peculiar regulation compared to the provirus (Gelderblom et al., 2008; Chan et al., 2016; Thierry et al., 2016). In this case, the faint viral production, under INSTI condition, could lead to a weak infection of newly cells providing newly complex formed by 2-LTR circles and IN in these cells. If these hypotheses are confirmed, 2-LTRc could be considered as a reservoir for HIV-1 integration and thus a molecule involved in pre-integration latency.

## Conclusion

Development of the INSTIs compounds is a great advance in treatment-naïve and experienced HIV-infected patients. Inclusion of INSTI in the regimen is considered as a first-line therapy for treatment-naïve infected patients. However, despite their efficacy to decrease the viral load, one must not only monitor the emergence of resistance mutations, but also take care of the presence of uDNA that could be a source of viral escape, either by integration of 2-LTRc or by expression of uDNA at the origin of basal replication. The quantitative importance of these uDNA forms under treatment with catalytic integrase inhibitors highlights the issue of reservoirs cells. In particular, the key DNA forms in latent reservoirs such as quiescent memory CD4-T cells, the only reservoir where long-term persistence of HIV-1 in patients receiving optimal antiretroviral therapy has been repeatedly described, could not be only integrated DNA but could involve uDNA. Treatment with a non-catalytical integrase inhibitor in the context of antiretroviral therapy could thus have different implications in terms of reservoirs.

These alternative pathways making use of uDNA to escape INSTI treatment emphasizes the need to understand the nature of the viral DNA forms in the various reservoirs cells. This knowledge would fuel the research developing curative strategies that cannot bypass the question of reservoirs.

## Author Contributions

OD initiated the project. ET, ED, and OD wrote the article. All the authors reviewed the final version of the manuscript prior to submission for publication.

## Conflict of Interest Statement

The authors declare that the research was conducted in the absence of any commercial or financial relationships that could be construed as a potential conflict of interest.
